# Formation Tracking Control for Multi-Agent Networks with Fixed Time Convergence via Terminal Sliding Mode Control Approach

**DOI:** 10.3390/s21041416

**Published:** 2021-02-18

**Authors:** Guang-Hui Xu, Meng Li, Jie Chen, Qiang Lai, Xiao-Wen Zhao

**Affiliations:** 1 Hubei Key Laboratory for High-efficiency Utilization of Solar Energy and Operation Control of Energy Storage System, School of Electrical and Electronics Engineering, Hubei University of Technology, Wuhan 430068, China; xgh@hbut.edu.cn (G.-H.X.); 101810207@hbut.edu.cn (M.L.); 2 School of Science, Hubei University of Technology, Wuhan 430068, China; 3 School of Electrical and Automation Engineering, East China Jiaotong University, Nanchang 330013, China; chaos1963@ecjtu.edu.cn; 4 School of Mathematics, Hefei University of Technology, Hefei 230009, China; zhaoxiaowen@hfut.edu.cn

**Keywords:** multi-agent networks, fixed time convergence, formation tracking control, sliding mode control

## Abstract

This paper investigates formation tracking control for multi-agent networks with fixed time convergence. The control task is that the follower agents are required to form a prescribed formation within a fixed time and the geometric center of the formation moves in sync with the leader. First, an error system is designed by using the information of adjacent agents and a new control protocol is designed based on the error system and terminal sliding mode control (TSMC). Then, via employing the Lyapunov stability theorem and the fixed time stability theorem, the control task is proved to be possible within a fixed time and the convergence time can be calculated by parameters. Finally, numerical results illustrate the feasibility of the proposed control protocol.

## 1. Introduction

Coordinated control for multi-agent networks has been studied with more and more attention recently owing to its diversified application value, such as consensus [1,2,3], formation [4,5,6], containment [7,8], flocking [9,10] and tracking [11,12] control problems. In [13], the synchronization control problem of multi-agent networks is solved by using chaos theory and the result is applied to the coupling circuits. It is generally appreciated that consensus, the purpose of which is to control a set of agent states to reach an agreement, is a fundamental and important problem. The consensus issues include consensus without leader [14,15] and leader-follower consensus [16,17]. In [18], the consensus problem is extended to the sensor network and a distributed particle filter is proposed. With the development of technology, the consistent state of all agents can no longer meet people’s needs to solve more complex problems such as drone formation performance, multi-robot collaborative work and so forth. Based on the consensus control problem, the formation control problem is proposed. Generally speaking, formation refers to the position of multi-agent achieving a specific geometric formation by using local information. Formation control is an indispensable part of coordination control problems of multiple agent networks.

Recently, the problems of formation control have been investigated extensively and used in many practical networks, such as unmanned aerial vehicles [19] and multi-robot networks [20]. In particular, based on the consensus tracking control problem, experts proposed formation tracking control, which has led many scholars to conduct research in this area because it is more suitable for practical engineering applications. In [21], the formation control problem is studied under the condition of agents’ communication topology diagram dynamics, in which the formation of agents changes over time. The leader-following formation problems for multi-agent networks have been researched in [22], where the communication delay is considered. The formation problem for double-integrator agent networks is resolved in [23] by using output feedback information. A new formation control protocol by using Stress-matrix was proposed in [24], in which formations composed of multi-agent systems can be scaled down or enlarged freely. In [25], the multi-agent network is divided into many sub-networks, and the communication weight between the sub-groups is zero. A new control protocol is designed to make it so that each sub-group can form a different formation to complete more complex tasks.

The aforementioned research on multi-agent control mostly based on asymptotic stability, which means that the desired task is achieved in infinite time. Moreover, when studying control problems of convergence time for multi-agent networks, the finite time convergence method is adopted to enhance the computational efficiency. Consensus control of higher-order multiple agent networks is solved in [26] by using the control method within finite-time convergence. In [27], finite-time synchronization control problem of fuzzy complex dynamics network is solved. Fu et al. [28] analyzed the leader-following consensus problems with finite-time convergence under two models of relative output obtainable and relative state measurable in directed communication graph and undirected communication graph, respectively. Through using the TSMC approach, Han et al. [29] realized finite-time three-dimensional formation for multi-agent networks while the leader’s control input is zero and non-zero. The formation containment tracking problem with finite time convergence for Euler-Lagrange networks with hierarchical leaders is studied in [30]. However, the convergence time of these research results needs to be calculated by the initial state of the network, which is not applicable in many practical situations.

Currently, the result of [31] shows sufficient conditions of fixed-time stability for non-linear systems by employing the TSMC approach and the convergence time can be calculated by the control protocol’s parameters. In [32], based on the TSMC approach, leader-following control protocols are proposed to deal with the consensus problem of double-integrator agent networks with fixed time convergence in the case of a leader agent’s control input is unknown. A TSMC differentiator observer is proposed in [33] to design leader-following control protocols of integrator dynamic systems based on output feedback. In [34], the synchronization tracking control of the manipulator is realized for fixed-time convergence.

Based on the above analysis, the problem we need to solve is distributed formation tracking of multiple agent networks with fixed time convergence. The main contributions of this article are as follows: first through the TSMC approach, a control protocol based on the error system is designed. Second, a group of agents can complete the formation designed according to the actual requirements under the designed control protocol, and the entire multi-agent network follows the leader to do synchronous motion in a fixed time. Finally, the convergence time of the system from the initial state to the steady state can be calculated by the parameters of the control protocol. The results in [32] can be seen as the results of this paper if the offset of the formation is zero.

The rest of this article is structured as follows. In Section 2, some nations of algebraic graph theory, the system description and the control problem description are stated. In Section 3, by employing the TSMC approach to design a new control protocols control protocol, the formation tracking problem of multi-agent networks with fixed time convergence is solved. In Section 4, numerous simulation examples are presented. Finally, conclusions and further works are proposed in Section 5.

The following concepts are used in this paper: let ℛ represent the n-dimensional Euclidean space. 0, 1 and I are the zero vector, unit vector and identity matrix, respectively, where the dimensions will be given in the article. Let $diag\left\{c_{1},c_{2},\ldots c_{n}\right\}$ indicate diagonal matrix with scalar entries $c_{i}$. For any given matrix H, $H^{T}$ represent its transpose. The mathematics symbol ⊗ is the Kronecker product.

## 2. Preliminaries

### 2.1. Graph Theory

The communication topology of a multi-agent network is described by an algebraic directed graph $𝒢=\left(𝒱,E\right)$, in which $𝒱=\left\{1,2,\ldots ,n\right\}$ is the set of agents, E⊆𝒱×𝒱 is the edges set. An edge $\left(j,i\right)\in E$ if ith agent can access the information of jth agent directly, but not vice versa, and in this case agent j is the neighbor of agent i. The neighbors set about agent i can be described as ${𝒩}_{i}=\left\{j\in 𝒱/\left(i,j\right)\in E,j\neq i\right\}$. The weighted adjacency matrix is described as $W=\left[w_{ij}\right]\in ℛ$, in which $w_{ij}>0$ if $j\in {𝒩}_{i}$, otherwise $w_{ij}=0$. It is worth noting that there is no self-loop in the graph of this paper, namely, $w_{ii}=0$. The degree matrix is described as a diagonal matrix $D=diag\left\{d_{1},d_{2},\ldots d_{n}\right\}\in ℛ$ with the diagonal scalar entries $d_{i}=\sum_{j=1}^{n}w_{ij},i=1,2,\ldots ,n$. The Laplacian matrix L of graph 𝒢 is defined as L=D−W. Moreover, a diagonal matrix is described as $\prod =diag\left\{\pi_{1},\ldots ,\pi_{n}\right\}\in ℛ$, where $\pi_{i}>0$ if the agent i can access the information about leader directly, otherwise $\pi_{i}=0$.

### 2.2. Problem Description

In this part, a corresponding agent networks include N+1 agents and the leader agent is described as 0 and follower agents are described as 1,2,…,N. For further analysis, some definitions and assumptions are presented as follows.

**Definition** **1.** *A multi-agent network is composed of the leader and followers due to the specific task needs. Leader is required to provide a reference trajectory for followers, so it only sends information to some of the follower agents. The follower agents are required to achieve prescribed formation, and formation tracks the leader’s status simultaneously and they can interact with their neighbor follower agents but only part of them can interact with the leader agent.*

**Assumption** **1.** *For directional communication topology* 𝒢 *between agents, there is a directional spanning tree, in which the leader represents the root node.*

**Remark** **1.** *Based on the results in [35], the matrix described as* $\bar{L}=L+W$ *is invertible and can be confirmed if Assumption 1 is held.*

The dynamic model of the leader agent is defined as:

(1) $$\left\{\begin{array}{l} {\dot{x}}_{0}\left(t\right)=v_{0}\left(t\right),\\ {\dot{v}}_{0}\left(t\right)=u_{0}\left(t\right), \end{array}\right.$$

where $x_{0}\in ℛ$, $v_{0}\in ℛ$ and $u_{0}\in ℛ$ represent the position, velocity and control input of the leader.

The dynamic model of the follower agents are defined as

(2) $$\left\{\begin{array}{l} {\dot{x}}_{i}\left(t\right)=v_{i}\left(t\right),\\ {\dot{v}}_{i}\left(t\right)=u_{i}\left(t\right),i=1,2,\ldots ,N, \end{array}\right.$$

where $x_{i}\in ℛ$, $v_{i}\in ℛ$ and $u_{i}\in ℛ$ represent the position, velocity and control input of followers.

**Definition** **2.** *(formation tracking with fixed-time convergence). For a set of agents, given an arbitrary bounded initial position, if*

(3) $$\left\{\begin{array}{l} \underset{x\to T}{\lim}\left(x_{i}\left(t\right)-\xi_{i}-x_{0}\left(t\right)\right)=0,\;\\ \underset{x\to T}{\lim}\left(v_{i}\left(t\right)-v_{0}\left(t\right)\right)=0, \end{array}\right.$$

and

(4) $$\left\{\begin{array}{l} x_{i}\left(t\right)-\xi_{i}-x_{0}\left(t\right)=0,\;\\ v_{i}\left(t\right)-v_{0}\left(t\right)=0,\;i=1,2,\ldots ,N,\; \end{array}\right.$$

as t≥T, then formation tracking control of multi-agent networks with fixed time convergence is accomplished and convergence time T satisfying $T<T_{\max}$ and $\xi_{i}$ is the desired geometric formation.

**Lemma** **1.** *[36] Consider an integrator system*

(5) $$\dot{y}=-\alpha y^{\frac{m}{n}}-\beta y^{\frac{p}{q}},\;y\left(0\right)=y_{0},$$

where α>0, β>0, m, n, p and q are positive odd integers satisfying $m > n$ and p<q. The equilibrium of Equation (5) is globally fixed-time stable with settling time T bounded by

(6) $$T<T_{\max}:=\frac{1}{\alpha }\frac{n}{m-n}+\frac{1}{\beta }\frac{q}{q-p}.$$

Moreover, if $\varepsilon =\left[q\left(m-n\right)\right]/\left[n\left(q-p\right)\right]\leq 1$, then a less conservation estimation of the settling time can be obtained instead as

(7) $$T<T_{\max}:=\frac{q}{q-p}\left(\frac{1}{\sqrt{\alpha \beta }}\tan^{-1}\sqrt{\frac{\alpha }{\beta }}+\frac{1}{\alpha \varepsilon }\right).$$

## 3. Fixed-Time Formation Tracking Control

In this part, a new control protocol is designed to solve the problem of distributed formation tracking control with fixed time convergence based on the relevant information of neighboring agents. Different from the existing results, under the effect of the control protocol proposed in this article, the time for the follower to achieve formation control is a fixed time that can be calculated by the control parameters. The content of this section is arranged as follows: firstly, the closed-loop error system and sliding mode variables are designed based on the neighbor’s state information, and then the new control protocol is designed by using the constructed mode variables. Moreover, strict and complete evidence will be presented to demonstrate the adequacy of the proposed control protocol. Finally, the consensus tracking control problem with fixed time convergence will be solved similarly to the method of the formation tracking control problem with fixed time convergence.

### 3.1. Formation Control for Multi-Agent Networks with Fixed Time Converges

Before studying formation control problems, the error system is first proposed for follower agents:

(8) $$\left\{\begin{array}{c} e_{ix}\left(t\right)=\sum_{j=1}^{N}w_{ij}\left[\left(x_{i}\left(t\right)-\xi_{i}\right)-\left(x_{j}\left(t\right)-\xi \right)\right]\\ +\pi_{i}\left(x_{i}\left(t\right)-\xi_{i}-x_{0}\left(t\right)\right).\\ e_{iv}\left(t\right)=\sum_{j=1}^{N}w_{ij}\left(v_{i}\left(t\right)-v_{j}\left(t\right)\right)+\pi_{i}\left(v_{i}\left(t\right)-v_{0}\right). \end{array}\right.$$

It can be seen from Definition 2, when the error system (8) converges to zero, the formation control task is implemented.

Sliding mode variables are designed according to the error system as follows:

(9) $$\begin{array}{l} s_{i}\left(t\right)=e_{ix}+{\left[{\left(\alpha e_{ix}^{\left(m_{1}/n_{1}\right)-\left(p_{1}/q_{1}\right)}+\beta \right)}^{-1}e_{iv}\right]}^{q_{1}/p_{1}}\\ =e_{ix}+{\left(\varphi e_{iv}\right)}^{q_{1}/p_{1}}, \end{array}$$

where $\phi =1/\left(\alpha e_{ix}^{m_{1}/n_{1}-p_{1}/q_{1}}+\beta \right)>0$, α>0, β>0 and parameters $m_{1}$, $n_{1}$, $p_{1}$, $q_{1}$ are positive odd integers satisfying $m_{1}>n_{1}$, $p_{1}<q_{1}$ and $m_{1}/n_{1}-p_{1}/q_{1}>0$.

**Remark** **2.** *From the structure of the sliding surface (9), when the system reaches the sliding surface, that is, when* $s_{i}=0$ *, we can get the following equation:*

(10) $$\;e_{iv}={\dot{e}}_{ix}=-\alpha e_{ix}^{m_{1}/n_{1}}-\beta e_{ix}^{p_{1}/q_{1}}.$$

Equation (10) meets the requirements of Lemma 1, then the position error $e_{ix}$ can converge to zero in a fixed-time.

Hence, for the multi-agent networks Equations (1) and (2), a novel control protocol employing nonlinear TSMC is proposed as:

(11) $$\begin{array}{c} u_{i}={\left(\sum_{j=1}^{N}w_{ij}+\pi_{i}\right)}^{-1}\left[\sum_{j=1}^{N}w_{ij}u_{j}+\pi_{i}u_{0}\right.+\alpha \left(\frac{m_{1}}{n_{1}}-\frac{p_{1}}{q_{1}}\right)\varphi {\left(e_{ix}\right)}^{\frac{m_{1}}{n_{1}}-\frac{p_{1}}{q_{1}}-1}e_{iv}^{2}\\ \;-\frac{p_{1}}{q_{1}}\varphi^{-\frac{q_{1}}{p_{1}}}{\left(e_{iv}\right)}^{2-\frac{q_{1}}{p_{1}}}-\frac{p_{1}}{q_{1}}\varphi^{-\frac{q_{1}}{p_{1}}}{\left(e_{iv}\right)}^{1-\frac{q_{1}}{p_{1}}}\left.\left(\gamma {\left(s_{i}\right)}^{\frac{m_{2}}{n_{2}}}+\lambda {\left(s_{i}\right)}^{\frac{p_{2}}{q_{2}}}\right)\right], \end{array}$$

where γ>0, λ>0, $m_{2}>n_{2}$ and $m_{2}$, $n_{2}$ are positive odd integers.

**Theorem** **1.** *For multi-agent networks Equations (1) and (2), given an arbitrary bounded initial position and Assumption 1 is held, the control problem of formation tracking with fixed-time convergence is solved under the control protocol (11), in which the convergence time* T *required for the system to reach stability is described as follows:*

(12) $$T<T_{\max}=T_{1}+T_{2}=\frac{n_{1}}{\alpha \left(m_{1}-n_{1}\right)}+\frac{q_{1}}{\beta \left(q_{1}-p_{1}\right)}+\frac{n_{2}}{\gamma \left(m_{2}-n_{2}\right)}+\frac{q_{2}}{\lambda \left(q_{2}-p_{2}\right)}.$$

**Proof** **of** **Theorem** **1.** *The proof process is proposed in this part, let*$e_{x}={\left[e_{1x}^{T},e_{2x}^{T},\ldots ,e_{Nx}^{T}\right]}^{T}$, $e_{v}={\left[e_{1v}^{T},e_{2v}^{T},\ldots ,e_{Nv}^{T}\right]}^{T}$, *and*$u={\left[u_{1}^{T},u_{2}^{T},\ldots ,u_{N}^{T}\right]}^{T}$*be the position error, velocity error and control input vectors, respectively. Then the time derivatives of the formation tracking errors can be obtained in the following form:*

(13) $$\left\{\begin{array}{l} {\dot{e}}_{x}\left(t\right)=e_{v}\left(t\right).\\ {\dot{e}}_{v}\left(t\right)=\left(\bar{L}⊗I\right)u\left(t\right)-\prod 1⊗u_{0}\left(t\right). \end{array}\right.$$

Taking the time derivative of sliding mode vector (9) yield

(14) $${\dot{s}}_{i}\left(t\right)=e_{v}+\frac{q_{1}}{p_{1}}{\left(\varphi e_{v}\right)}^{\frac{q_{1}}{p_{1}}-1}\left[-\alpha \left(\frac{m_{1}}{n_{1}}-\frac{p_{1}}{q_{1}}\right)\varphi^{2}e_{x}^{\frac{m_{1}}{n_{1}}-\frac{p_{1}}{q_{1}}-1}e_{v}^{2}+\varphi {\dot{e}}_{v}\right].$$

Let $s=\left[s_{1}^{T},s_{2}^{T},\ldots ,s_{N}^{T}\right]$. Then, the proposed control protocol $u$ could be rewritten as:

(15) $$\begin{array}{l} u=\left({\bar{L}}^{-1}⊗I\right)\left[\prod 1⊗u_{0}+\alpha \left(\frac{m_{1}}{n_{1}}-\frac{p_{1}}{q_{1}}\right)\varphi {\left(e_{x}\right)}^{\frac{m_{1}}{n_{1}}-\frac{p_{1}}{q_{1}}-1}e_{v}^{2}\right.\\ -\frac{p_{1}}{q_{1}}\varphi^{-\frac{q_{1}}{p_{1}}}{\left(e_{v}\right)}^{2-\frac{q_{1}}{p_{1}}}-\frac{p_{1}}{q_{1}}\varphi^{-\frac{q_{1}}{p_{1}}}{\left(e_{v}\right)}^{1-\frac{q_{1}}{p_{1}}}\left.\left(\gamma s^{\frac{m_{2}}{n_{2}}}+\lambda s^{\frac{p_{2}}{q_{2}}}\right)\right]. \end{array}$$

Lyapunov function $V_{2}=\left(1/2\right)s^{T}\left(t\right)s\left(t\right)$ is constructed by using the sliding mode variable, whose derivative with respect to time is

(16) $$\begin{array}{l} {\dot{V}}_{2}\left(t\right)=s^{T}\left(t\right)\dot{s}\left(t\right)\\ =s^{T}\left(t\right)\left\{e_{v}+\frac{q_{1}}{p_{1}}{\left(\varphi e_{v}\right)}^{\frac{q_{1}}{p_{1}}-1}\right.\cdot \left.\left[\varphi {\dot{e}}_{v}-\alpha \left(\frac{m_{1}}{n_{1}}-\frac{p_{1}}{q_{1}}\right)\varphi^{2}{\left(e_{x}\right)}^{\frac{m_{1}}{n_{1}}-\frac{p_{1}}{q_{1}}-1}e_{v}^{2}\right]\right\}. \end{array}$$

By using Equations (13) and (14), we get

(17) $$\begin{array}{l} {\dot{V}}_{2}\left(t\right)=s^{T}\left(t\right)\left(-\gamma s^{\frac{m_{2}}{n_{2}}}-\lambda s^{\frac{p_{2}}{q_{2}}}\right)\\ \leq -\gamma s^{\frac{m_{2}+n_{2}}{n_{2}}}-\lambda s^{\frac{p_{2}+q_{2}}{q_{2}}}\\ \leq -\gamma {\left(s^{2}\right)}^{\frac{m_{2}+n_{2}}{2n_{2}}}-\lambda {\left(s^{2}\right)}^{\frac{p_{2}+q_{2}}{2q_{2}}}\\ =-\gamma {\left(2V_{2}\right)}^{\frac{m_{2}+n_{2}}{2n_{2}}}-\lambda {\left(2V_{2}\right)}^{\frac{p_{2}+q_{2}}{2q_{2}}}. \end{array}$$

If $V_{2}\neq 0$, then let $y_{2}=\sqrt{2V_{2}}$ and ${\dot{y}}_{2}={\dot{V}}_{2}/\sqrt{2V_{2}}$. We can rewrite Equation (17) as

(18) $${\dot{y}}_{2}\left(t\right)=-\gamma {\left(y_{2}\right)}^{\frac{m_{2}}{n_{2}}}\left(t\right)-\lambda {\left(y_{2}\right)}^{\frac{p_{2}}{q_{2}}}\left(t\right).$$

By Lemma 1, we can obtain $\lim_{t\to t_{2}}V_{2}\left(s_{i}\right)=0$; here, the convergence time $t_{2}$ is defined as $t_{2}<T_{2}$.

At this point, the system reaches the sliding mode surface, that is, the sliding mode variable $s_{i}=0$. The following equation can be obtained from Remark 2.

(19) $${\dot{e}}_{ix}=-\alpha {\left(e_{ix}\right)}^{\frac{m_{1}}{n_{1}}}-\beta {\left(e_{ix}\right)}^{\frac{p_{1}}{q_{1}}}.$$

Consider Lyapunov function $V_{1}=\left(1/2\right)e_{ix}^{T}e_{ix}$ and its time derivative is

(20) $$\begin{array}{l} {\dot{V}}_{1}=-\alpha {\left(e_{xi}\right)}^{\frac{m_{1}+n_{1}}{n_{1}}}-\beta {\left(e_{xi}\right)}^{\frac{p_{1}+q_{1}}{q_{1}}}\\ \leq -\alpha {\left(2V_{1}\right)}^{\frac{m_{1}+n_{1}}{2n_{1}}}-\beta {\left(2V_{1}\right)}^{\frac{p_{1}+q_{1}}{2q_{1}}}. \end{array}$$

If $V_{1}\neq 0$, then let $y_{1}=\sqrt{2V_{1}}$ and ${\dot{y}}_{1}={\dot{V}}_{1}/\sqrt{2V_{1}}$. We can rewrite Equation (20) as

(21) $${\dot{y}}_{1}\left(t\right)=-\alpha y_{1}^{\frac{m_{1}}{n_{1}}}\left(t\right)-\beta y_{1}^{\frac{p_{1}}{q_{1}}}\left(t\right).$$

Equation (21) satisfies lemma 1, so we know that $\lim_{t\to t_{1}}V_{1}\left(e_{ix}\right)=0$ with setting time $t_{1}$ is bounded by $t_{1}<T_{1}$. Therefore, it can be proved that under the control protocol (11), the multi-agent networks with fixed time convergence can complete prescribed formation and its convergence time $T<T_{\max}=T_{1}+T_{2}$.

**Remark** **3.** *A new multi-agent network formation control algorithm with fixed time convergence is designed in this paper, where the sliding surface based on the velocity error system and the position error system is used to design the above protocol (11). From the perspective of the construction of the control protocol, the choice of control parameters must follow the requirements of Lemma 1, otherwise it will cause singularity.*

**Remark** **4.** *The main conclusion of this paper is given in the form of Theorem 1. In the process of the proof of theorem, for the convenience of recording, the state equation of the system is rewritten into a matrix form. The matrix knowledge used in the calculation is not complicated, only the basic operation rules of the matrix, the Kronecker product operation rules and the matrix eigenvalue analysis are required. These calculation rules can be found in the literature [37].*

**Remark** **5.** *Lemma 1 was used twice in the proof of Theorem 1, so the fixed convergence time is* $T<T_{\max}=T_{1}+T_{2}$ *. We can simplify the calculation of the final convergence time as* $T<T_{\max}=2T_{1}$ *, if the parameter satisfies* $m_{1}=m_{2}$ *,* $n_{1}=n_{2}$ *,* α=γ *,* β=λ *,* $p_{1}=p_{2}$ *and* $q_{1}=q_{2}$ *in the control protocol (11).*

**Remark** **6.** *Compared with the work of Li et al. [29], the sliding mode control method is used to achieve multi-agent network formation tracking control, but the conclusions of this paper are more accurate. The system convergence time is a specific value that can be calculated by parameters. However, the results of Li et al. show the system convergence time is a finite value related to the initial state of the system, which cannot be calculated by the control parameters.*

### 3.2. Formation Control for Multi-Agent Networks with Fixed Time Converges

In the next part, a special case is analyzed, where formation offset $\xi_{i}$ is zero. In this case, all the agents achieve consensus and track the leader’s trajectory simultaneously. The error system for follower agents is rewritten as follows.

(22) $$\left\{\begin{array}{l} \varepsilon_{ix}\left(t\right)=\sum_{j=1}^{N}w_{ij}\left[x_{i}\left(t\right)-x_{j}\left(t\right)\right]+\pi_{i}\left(x_{i}\left(t\right)-x_{0}\left(t\right)\right).\\ \varepsilon_{iv}\left(t\right)=\sum_{j=1}^{N}w_{ij}\left(v_{i}\left(t\right)-v_{j}\left(t\right)\right)+\pi_{i}\left(v_{i}\left(t\right)-v_{0}\right). \end{array}\right.$$

Based on the error system (22), the sliding mode variable Equation (9) was rewritten into a new sliding mode format as follows.

(23) $$\begin{array}{l} {\tilde{s}}_{i}\left(t\right)=\varepsilon_{ix}+{\left[{\left(\alpha \varepsilon_{ix}^{\left(m_{1}/n_{1}\right)-\left(p_{1}/q_{1}\right)}+\beta \right)}^{-1}\varepsilon_{iv}\right]}^{q_{1}/p_{1}}\\ =\varepsilon_{ix}+{\left(\tilde{\varphi }\varepsilon_{iv}\right)}^{q_{1}/p_{1}}, \end{array}$$

where $\tilde{\phi }=1/\left(\alpha \varepsilon_{ix}^{\left(m_{1}/n_{1}\right)-\left(p_{1}/q_{1}\right)}+\beta \right)>0$ and the parameters are the same as Equation (9).

Similarly, based on the error system Equation (21) and the sliding mode variable Equation (22), a non-linear control protocol with fixed time convergence is desired as follows.

(24) $$\begin{array}{c} {\tilde{u}}_{i}={\left(\sum_{j=1}^{N}w_{ij}+\pi_{i}\right)}^{-1}\left[\sum_{j=1}^{N}w_{ij}u_{j}+\pi_{i}u_{0}\right.+\alpha \left(\frac{m_{1}}{n_{1}}-\frac{p_{1}}{q_{1}}\right)\tilde{\varphi }{\left(\varepsilon_{ix}\right)}^{\frac{m_{1}}{n_{1}}-\frac{p_{1}}{q_{1}}-1}\varepsilon_{iv}^{2}\\ \;-\frac{p_{1}}{q_{1}}{\tilde{\varphi }}^{-\frac{q_{1}}{p_{1}}}{\left(\varepsilon_{iv}\right)}^{2-\frac{q_{1}}{p_{1}}}-\frac{p_{1}}{q_{1}}{\tilde{\varphi }}^{-\frac{q_{1}}{p_{1}}}{\left(\varepsilon_{iv}\right)}^{1-\frac{q_{1}}{p_{1}}}\left.\left(\gamma {\left({\tilde{s}}_{i}\right)}^{\frac{m_{2}}{n_{2}}}+\lambda {\left({\tilde{s}}_{i}\right)}^{\frac{p_{2}}{q_{2}}}\right)\right], \end{array}$$

where the parameters are the same as Equation (11).

**Corollary** **1.** *For multi-agent networks Equations (1) and (2), given an arbitrary bounded initial position and when Assumption 1 is held, the control problem of consensus tracking with fixed-time convergence is solved under the control protocol (24), in which convergence time* T *is described as:*

(25) $$T<T_{\max}=T_{1}+T_{2}=\frac{n_{1}}{\alpha \left(m_{1}-n_{1}\right)}+\frac{q_{1}}{\beta \left(q_{1}-p_{1}\right)}+\frac{n_{2}}{\gamma \left(m_{2}-n_{2}\right)}+\frac{q_{2}}{\lambda \left(q_{2}-p_{2}\right)}.$$

The proof steps are similar to the previous ones and are omitted here.

**Remark** **7.** *Note that the conditions of the control protocol (24) are similar to those of protocol (11). Therefore, the control protocol can only be implemented under the condition of Assumption 1. In other words, the leader’s status information can be directly or indirectly transmitted to all agents.*

## 4. Simulation Studies

**Figure 1:**
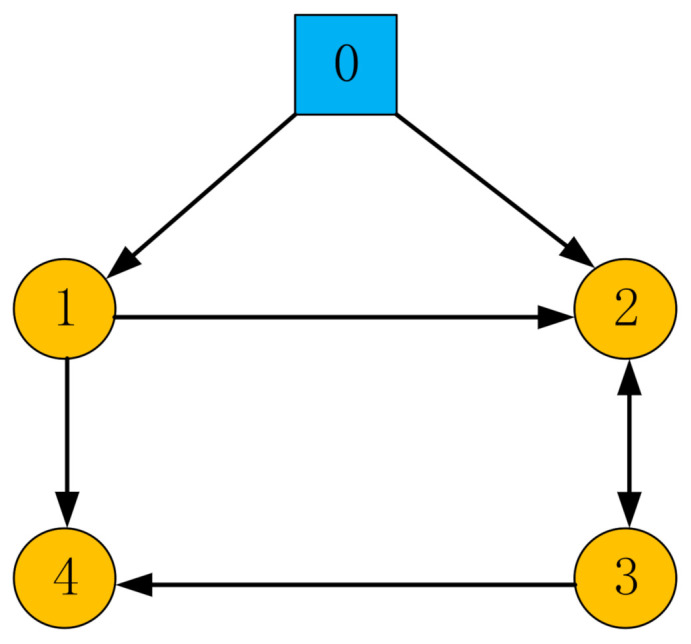
The communication topology of multi-agent network.

In this part, the feasibility of the protocol mentioned above is proved by numerical results with MATLAB software. Without loss of generality, consider a network of five agents (node marked 0 indicates the leader agent, others indicate follower agents) and the directed communication topology diagram designed under the condition of satisfying Assumption 1, as shown in Figure 1, where the arrow points to indicate the direction of information flow between agents, and the two-way arrow indicates the exchange of information between agents. The numerical simulation example is to verify in a two-dimensional plane whether the formation of fixed time convergence can be achieved under the control protocol designed in this paper.

**Figure 2:**
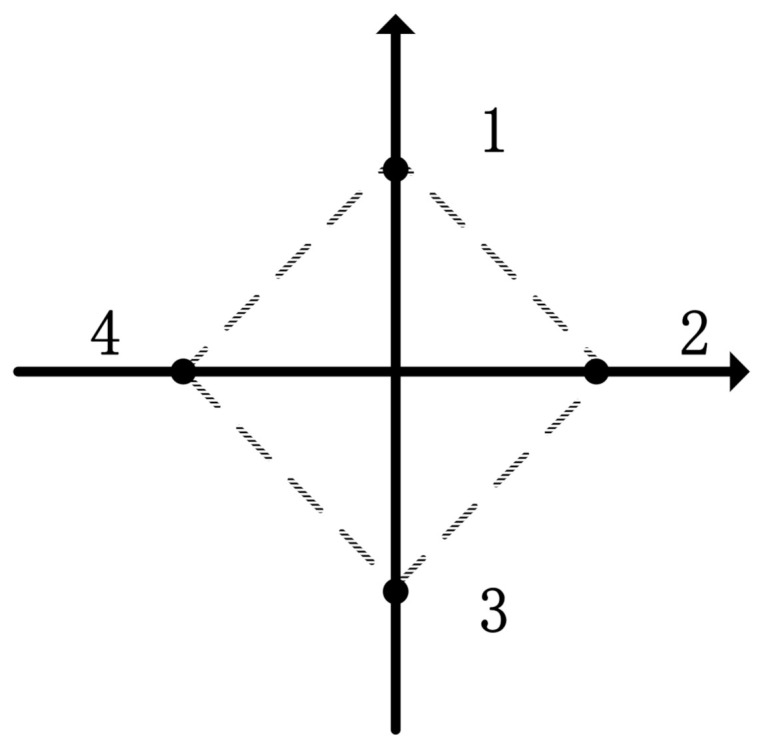
The communication topology of multi-agent network.

The rhombus described in Figure 2 is the prescribed geometric formation for follower agents with the leader as the origin and its local coordinates as $\left(0,0.4\right)$, $\left(0.4,0\right)$, $\left(0,-0.4\right)$ and $\left(-0.4,0\right)$. 

In order to simplify the study, set all weight values to one, diagonal matrix ∏ is $\prod =diag\left(1,1,0,0\right)$ and the corresponding Laplacian matrix L and $\bar{L}$ designed as:

$$L=\left[\begin{array}{cccc} 0 & 0 & 0 & 0\\ -1 & 2 & -1 & 0\\ 0 & -1 & 1 & 0\\ -1 & 0 & -1 & 2 \end{array}\right],\bar{L}=\left[\begin{array}{cccc} 1 & 0 & 0 & 0\\ -1 & 3 & -1 & 0\\ 0 & -1 & 1 & 0\\ -1 & 0 & -1 & 2 \end{array}\right].$$

The time-varying trajectory of the leader agent in a two-dimensional plane is designed as follows. Both the abscissa *x*-axis and ordinate *y*-axis are represented by trigonometric functions.

$$\left\{\begin{array}{l} x_{0}=\left[2.4+0.8\cos\left(0.4t\right),1.4+\sin\left(0.4t\right)\right]\\ v_{0}=\left[-0.32\sin\left(0.4t\right),0.4\cos\left(0.4t\right)\right]\\ u_{0}=\left[-0.128\cos\left(0.4t\right),0.16\sin\left(0.4t\right)\right]. \end{array}\right.$$

The design parameters α=β=1, γ=λ=2, $m_{1}=9$, $n_{1}=7$, $p_{1}=5$, $q_{1}=7$, $m_{2}=11$, $n_{2}=9$, $p_{2}=7$ and $q_{2}=9$ are set for protocol (11), which meet the requirements of Lemma 1. Then the fixed-time T can be calculated as 11.5 s.

**Figure 3:**
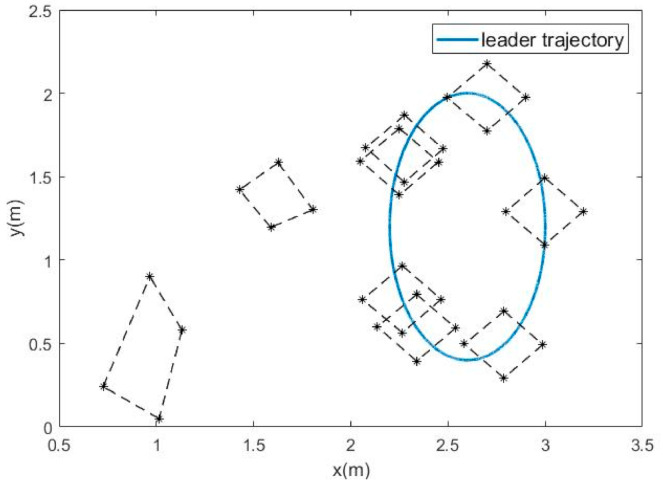
Motion trajectories of agents.

**Figure 4:**
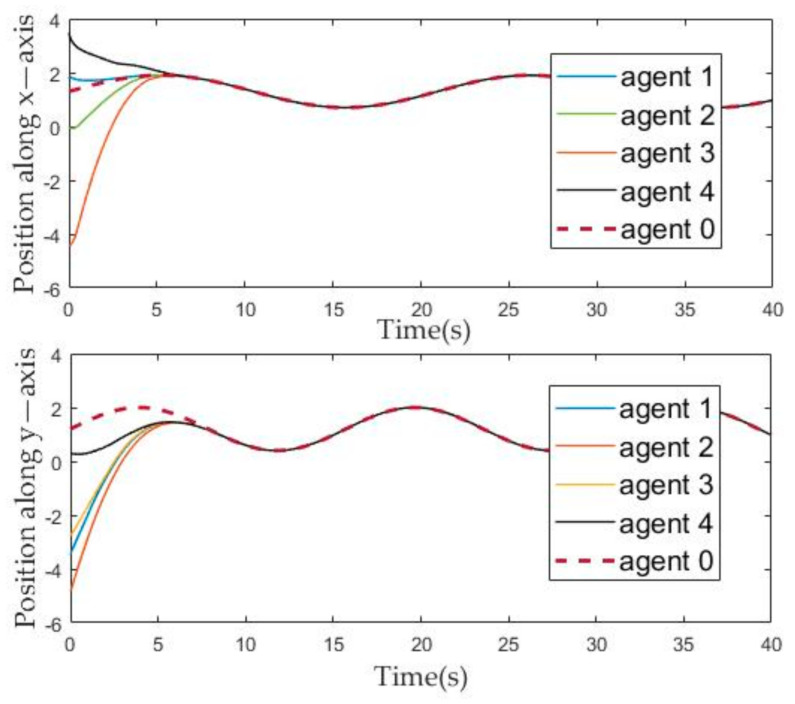
The evolution of the agents’ position state over time.

**Figure 5:**
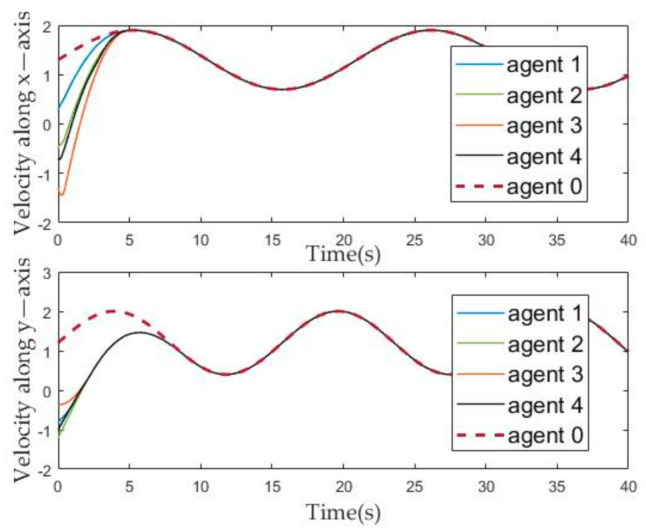
The evolution of the agents’ velocity state over time.

**Figure 6:**
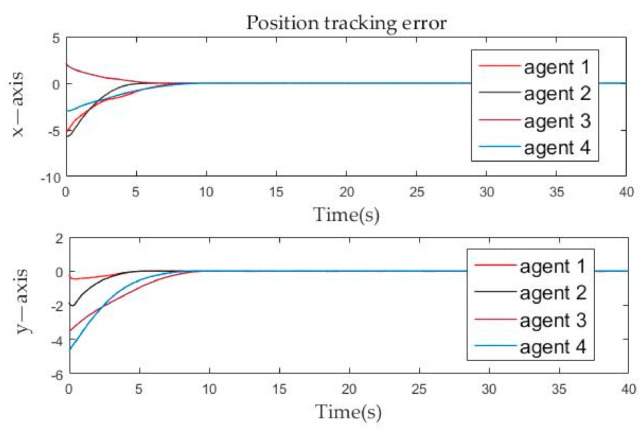
Position tracking errors evolution of follower agents over time.

**Figure 7:**
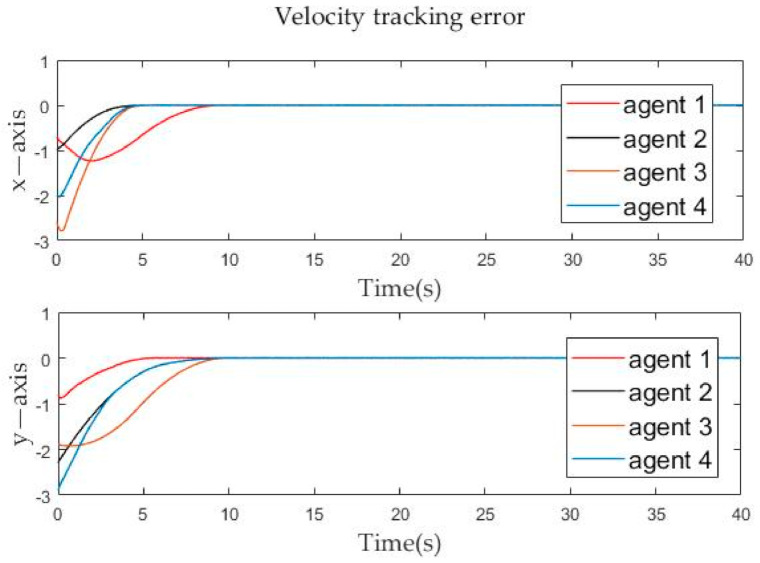
Velocity tracking errors evolution of follower agents over time.

The numerical simulation results in Figure 3, Figure 4, Figure 5, Figure 6 and Figure 7 show that the formation tracking control problem for multiple agent networks (1) and (2) is achieved by employing the novel protocol (11). Figure 3 records the motion trajectory of agents at 3 s, 6 s, 10 s, 13 s, 16 s, 19 s, 22 s, 23 s and 27 s, respectively. Follower agents start from the bottom left, track the leader and move in a clockwise direction with the leader and it is easy to see that all the follower agents accomplished the given geometric formation in about 10 s and then the geometric center of the formation moves synchronously with the leader.

Figure 4 and Figure 5 describe the change of the position and velocity of the agent over time from the *x*-axis and *y*-axis, respectively. In order to facilitate the observation of the effect of the position tracking of follower and leaders, we adjusted the position of each agent, that is, subtracted their corresponding offset from the position of each agent. Therefore, it can be seen from Figure 4 that the position evolution curve of the followers can coincide with the position evolution curve of the leader after about ten seconds. Similarly, it can be seen from Figure 5 that, after about ten seconds, the velocity curve of the follower can coincide with the leader.

The position tracking errors and velocity tracking errors between follower agents and leader agent are described in Figure 6 and Figure 7. It can be seen from the figure that whether it is the velocity error or the position error of the follower agents, it gradually approaches zero in the *x*-axis and *y*-axis directions, and becomes zero at about the tenth second. By Definition 2, it is obvious that both position and velocity are matched around the tenth second, which means that formation tracking is achieved. The calculated result from Theorem 1 is very close to the simulation result. Then the effectiveness of the control protocol is proven.

**Remark** **8.** *In [29], the formation tracking control with finite time convergence is studied by using sliding mode control, and the two numerical simulation examples given show that the system convergence time is 15 s and 18 s, respectively. It is worth noting that the convergence of the numerical simulation results in this article is 10 s, which means that the control protocol in this article is more effective. The system convergence time shown in the numerical simulation of [32] is not much different from that of this paper, but under the control protocol, it can only achieve consensus control. As we all know, consensus control for multi-agent networks is only a special case of formation control for multi-agent networks when the formation offset is below zero. Therefore, the results in [32] can be regarded as special cases of this article.*

## 5. Conclusions

The formation tracking control problem for multi-agent networks with fixed time convergence via the TSMC approach is studied in this paper. First, an error system employing the status information of neighboring agents has been used to design sliding mode variables and control protocols. Secondly, by using the Lyapunov stability theorem and the fixed time stability theorem, the protocol mentioned above has been proved to be correct. In addition, considering the special case where the formation offset is zero, consensus tracking problems of multiple agent networks with fixed time convergence has been solved. Finally, the example of numerical simulation has shown that the theoretical result is effective. Future work will focus on the predefined-time control of multi-agent networks and multi-robot systems.

## Data Availability

The data that support the findings of this study are available from the corresponding author, G.-H.X., upon reasonable request.
